# Supplementary material for: Patterns of Intron Gain and Loss in Fungi
Source: PLoS Biol. 2004 Nov 30;2(12):e422. doi: 10.1371/journal.pbio.0020422 (PMC532390; doi:10.1371/journal.pbio.0020422)
Supplement: Table S1 — Also available at http://genes.mit.edu/NielsenEtAl/. (4.3 MB ZIP). [file pbio.0020422.st001.zip › NielsenEtAl/html/672.html]

AN1378.1.NCU05498.1.MG00432.1.FG08359.1


```
 CLUSTAL W (1.82) Multiple Sequence Alignments - Introns Inserted


Sequence 1: AN1378.1	192 aa
Sequence 2: FG08359.1	178 aa
Sequence 3: MG00432.1	174 aa
Sequence 4: NCU05498.1	180 aa
Alignment Length: 201 aa
Number Identitical Residues: 80 aa
Alignment Score (without introns) 3838


MG00432.1 	---------MSEP--LTKVDSAVQGLSSSPKD-------TKSSAAARRASSAAASGVYNI
NCU05498.1	--------MSGEP--LTKVDSAVQGLEATSPP-------KESHIHKHRRASSSANGVMNI
FG08359.1 	---------MTEP--LSKVDSAVQGLSSSP---------PKEEKKTRRQSSAAAPGVYNI
AN1378.1  	MSLVDDHTDHTEHNELKKVDSAIGGLSISPKDEKASDKAEKADKKAHRRTSSQAEGVYNI
          	 :  .. :   * ..*.*****: **. :.  ..::..: :     :* :*: * ** **

MG00432.1 	KDLE1EQKIEIEIAKETQRTGW2KVNTSPSTVEDKEVLKKLLTTPPIKKIDLHFPLGLEV
NCU05498.1	NDLE~AQGTDLQIAKETQMLGW2KVNTSPTSIDDKDYLKVMLTTPPVKKIDLYFKTGLHV
FG08359.1 	NDLE1AEGIELELAPETQKTGW2KINKSSSTVEDAAILKLHLTKPPVKRIALHFPLGLEV
AN1378.1  	KELE1EKRIEITLPIETQKTGW2KLNTSPSTIEDKDILKLHLVNPPVKKIDLHFPLGLEV
          	::**  :  :: :. ***  ** *:*.*.::::*   **  *..**:*:* *:*  **.*

MG00432.1 	TARNLKGVTIKDAMDAIHKAYKKR0ADDELDQPYLAGFEWDKEECWTRFIVHLSKDS---
NCU05498.1	TARNLKGVTIRDAMDAIHKSFKKR0PDDELPEPYLKGFEWDKEESWTSLLVHLSTNAGP-
FG08359.1 	QARNLKGVTIKDALDAIHKAYKKR0SDDELDKPYLAGFEWDKEESWTQLIVHTQAQPTAT
AN1378.1  	TARNLKGVTIKDALDAIYKQFKKK0ADDELDKPYLAGFEWDKEECWTRLIVHQTKQ----
          	 *********:**:***:* :**: .**** :*** ********.** ::**   :    

MG00432.1 	---GVQANAGGKKKKKAAAEE---
NCU05498.1	---KPAGGSSKKKKKAAAAEEEAA
FG08359.1 	TLDGPSGGGGGKKKKNKKNEE---
AN1378.1  	---GPPAQSSGKKSKKKAKEEA--
          	      . .. **.*    **
```
